# Supplementary material for: Are carbon emissions trading and green financial instruments synergistic? -Comprehensive quantitative research based on content analysis
Source: PLoS One. 2024 Mar 7;19(3):e0298601. doi: 10.1371/journal.pone.0298601 (PMC10919601; doi:10.1371/journal.pone.0298601)
Supplement: S1 Data — (ZIP) [file pone.0298601.s001.zip › Data/地级市Z_F工作报告.zip!/╦─┤¿/╦∞─■╩╨/└┤╫╘╣1⁄2╓┌║┼í╛┬φ┐╦╩2╛▌═°í┐_2017.docx]

各位代表：

我代表市人民政府向大会报告工作，请予审查，并请市政协委员和其他列席同志提出意见。

一、突破千亿大关，“十三五”开局良好

2016年是实施“十三五”规划开局之年，也是冲刺千亿目标决战之年。面对错综复杂的经济形势，在市委的坚强领导下，在市人大、市政协的监督支持下，市政府团结和依靠全市人民，大力推进富民强市“七大提升行动”，经济总量迈上千亿台阶，经济社会发展取得了新成绩。全年实现地区生产总值1008.5亿元，同比增长9.1%；地方一般公共预算收入54.6亿元，增长13.8%；全社会固定资产投资1125.8亿元，增长11.3%；社会消费品零售总额470.4亿元，增长13.2%；城乡居民人均可支配收入达到26962元、12423元，分别增长7.8%、9.2%，主要经济指标增速位居全省前列，实现“十三五”良好开局。

（一）供需两侧同步发力，经济运行稳中向好

优化供给扶持实体。推进供给侧结构性改革，出台32条降本增效措施，全面落实“营改增”等减税降费政策，帮助企业解决用电、用气、用工等具体困难，实体经济持续向好。中小微企业银行贷款余额达到404.5亿元、增长15.9%，直接融资达到120.7亿元、增长49.2%，全年减轻企业负担16亿元。PPI从9月转正，结束了35个月的负增长，规上工业增加值增速从前5月的9.5%逐月回升至11.2%，规上工业企业利润总额增长11.5%。

重抓项目稳定投资。纵深推进项目攻坚，120个省市重大项目完成投资481亿元。创维西部产业园、四川蓬船灌区、唐家渡电航等项目相继开工，喜之郎、威斯腾铁路物流园、浪漫地中海、中环线等项目加快推进。争取中央预算内投资项目补助资金7.8亿元、国家专项建设基金41.1亿元，置换政府债券61.2亿元，PPP项目撬动民间投资51亿元。新增建设用地规划指标3600公顷，成功创建全国国土资源节约集约模范市。新建成110千伏遂西、乌木输变电工程。

拓展市场拉动内需。着力扩大消费需求，消费对经济增长的贡献率提高3.3个百分点。积极参加省级市场拓展“三大活动”，大力营销“遂宁鲜”“遂宁造”产品，实现销售金额56亿元。成功举办第九届遂宁观音文化节、观音湖荷花节等节会活动，推动万达广场等大型商业综合体开业。以新型城镇化激发内需潜力，城镇化率提高1.1个百分点，成为国家新型城镇化综合试点城市。实施“绿色城镇·幸福生活”项目35个，建成公园路人行天桥、渠河路北延线等“缓堵保畅”工程，开工建设城市地下综合管廊7.7公里，海绵城市试点年度综合考评全国第二。出台21条去库存措施，棚改货币化安置比例达到70%，商品住房库存下降13.3%。建成绿色城镇示范镇3个、幸福美丽新村278个。

（二）调转结合量质并举，产业结构优化升级

新兴产业提速扩量。锂电及新材料、电子信息等四大成长型工业总产值突破400亿元，占规上工业比重提高1个百分点。天齐锂业产值、税收分别增长74.3%和800%，基础锂盐产量居全球同类企业第三位。磨溪净化厂年处理天然气77.3亿方，处理规模居全国第二位。江淮汽车三类整车生产资质落户遂宁，整车下线1.6万辆。服务业增加值增长11%，增速持续领跑全省。物流产业增加值增长19.5%，远成西部物流园全面营运。旅游收入增长23.5%，成为“中国最美养生休闲旅游城市”，跻身全省旅游十强市。电商交易额增长30.4%，建成省级电商进农村综合示范县2个。

传统产业企稳回升。纺织服装、油气盐化工、食品饮料产业产值由降转增，分别增长3%、5.4%、11.1%。沱牌舍得完成战略重组，产值、税收分别增长38.1%和21.2%，盛马化工、美丰集团、南华纺织等企业恢复生产。农业平稳发展，增加值增长3.7%。新建成高标准农田16.3万亩，粮食总产实现“十连增”，肉类总产达31.6万吨。“三品一标”农产品达515个，认证数量居全省前列。

园区平台上档升级。“141工程”深入推进，园区工业主营业务收入占全市规上工业的82.6%。遂宁开发区获得国家新型工业化产业示范基地授牌，中国西部现代物流港成为全国首批示范物流园区，3个产业园区成为省级特色产业基地。五大农业园区引进农业产业化项目25个，新建成主导产业核心区3.3万亩，3个农业园区成为省级现代农业示范园区。大英文旅产业园成为省级文化产业示范基地。

（三）挖潜借力内外联动，发展动能加快转换

改革攻坚有突破。按照市委部署，实施重大改革任务252项，财金互动支持实体经济措施得到国务院督查组肯定，民营企业创办园区等7项改革经验被省委改革办推广。清理取消34项市级行政审批事项，明确38个市级部门责任清单。遂宁农交中心进入省统一规范化交易市场，完成土地流转交易8.5万亩，农村产权抵押贷款余额达到1.75亿元。不动产统一登记实现全域颁证。遂州信用联社成功改制为农商银行。完成机关公务用车改革。报业体制改革荣获中国报业创新成果三等奖。

开放合作上台阶。成功举办2016绿色经济遂宁会议、“物流两会”、西部金穗粮展会等重大活动，圆满完成第十六届西博会主题市参展任务、第四届科博会轮值市任务，顺利组织建市以来最大规模的侨商考察。引进中世物流等投资5亿元以上重大项目48个，到位市外国内资金436亿元，实现进出口总额4.1亿美元。川贵广—南亚国际物流大通道加快建设，珠海港驻遂宁办事处揭牌，遂德高速、南遂潼高速BOT建设模式获省政府批复同意。

创新创业见成效。推进大众创业万众创新，创建国家级众创空间2个，新增创业担保贷款5000余万元，新设立登记企业、新增注册资本金分别增长47.2%和50.3%。积极融入成德绵全面创新改革试验，7家企业进入军工领域。扎实开展国家知识产权试点城市创建工作，新增国家高新技术企业12家，遂宁物流产业技术研究院升级为省级研究院，大英聚能科技牵头成立四川首个石墨烯应用技术研究院。

（四）兜底补短共建共享，民生保障坚实有力

脱贫攻坚首战首胜。坚持长短结合，打出政策组合拳，如期实现66个贫困村摘帽、33472名贫困人口脱贫。突出联动推进，建立市、县领导联系贫困村制度，每户贫困家庭都有1名帮扶干部。突出产业就业帮扶，355家企业参与脱贫攻坚，产业帮扶1.26万人，就业帮扶5846人。突出住房改善，完成易地扶贫搬迁住房2852套，改造贫困户危房4219户。突出保障兜底，实施贫困学生15年基础教育免学费等政策，在全国首创联村卫生室建设模式，县域内住院费用控制在10%以内，率先在全省实现低保线和扶贫线“两线合一”。

民生实事全面完成。十项民生工程和20件民生大事全部兑现，民生支出达138亿元、占一般公共预算支出69.01%。实施教育“补短”工程，新建公办幼儿园17所，升级改造农村薄弱学校73所，高考一本上线人数、录取人数均创建市以来新高。稳步推进分级诊疗，城市公立医院全部取消药品加成，国家卫生城市通过复审。城镇登记失业率控制在4.14%以内，养老保险覆盖人数突破100万，医疗保险参保率达98.6%。新建通村公路136公里、改善农村公路226公里。四级公共文化服务体系基本建成，公共文化服务阵地全部免费开放。体育场馆免费或低收费开放达96.8万人次。

社会大局持续稳定。完善社会稳定风险评估机制，“12345”政府服务热线办理满意率达94.9%。大力实施农村“雪亮工程”，依法打击违法犯罪活动，治安秩序持续好转。安全生产四项指标大幅下降，连续14年未发生重特大安全生产事故，安全生产形势持续稳定。强力推进食品药品监管，有力保障了“舌尖上的安全”。PM10平均浓度下降幅度居全省第一，节能减排完成省定目标。

各位代表，过去一年，我们扎实开展“两学一做”学习教育，严格落实中央八项规定和省市十项规定，加强权力运行制约和监督，严厉惩治腐败。深化法治政府创建，全面实施行政决策合法性审查，完善行政应诉工作机制。集中开展软环境整治，全面取消非行政许可审批，即办件比例达到69%，办理提速达到99.5%。人大代表建议、政协委员提案办理满意及基本满意率达100%。国防动员、人防、民兵预备役建设、双拥、民族宗教、保密、档案、地方志和妇女儿童等工作扎实推进。

各位代表，过去一年，全市上下沉着应对经济下行压力，决战决胜千亿目标，各项事业进步明显，成绩取得实属不易。这是省委、省政府和市委坚强领导的结果，是市人大、市政协监督支持的结果，是广大干部群众拼搏实干的结果，是历届班子接力前行的结果。在此，我代表市人民政府，向全市人民，向人大代表、政协委员，向各民主党派、工商联、无党派人士、各人民团体，向历届老领导和所有关心支持遂宁发展的各界朋友表示衷心感谢并致以崇高敬意！

各位代表，总结去年工作，我们清醒看到，当前我市经济社会发展还存在诸多困难和问题。实体企业经营困难，资金、能源等要素成本依然较高，部分企业亏损严重。投资结构改善不足，新引进的大项目、好项目较少，民间投资占全社会固定资产投资比重较上年下降10.7个百分点。产业结构性矛盾突出，工业传统产业占比仍在70%左右，服务业占比分别比全国、全省平均水平低22.5和16.3个百分点。民生领域欠账较多，环境承载压力越来越大，交通拥堵等“城市病”日益突显，教育、医疗、养老等方面还存在不少短板，民间借贷等领域的问题还没有得到彻底解决。一些政府工作人员服务不到位，不担当、不作为等现象还没有根本改变。我们将牢记使命，以勇于担责的态度、事不避难的决心，正视问题，剖析原因，拿出对策，认真加以解决。

二、致力富民强市，加快发展弯道超车

今年是落实省委“加快发展、弯道超车”要求的关键之年，也是走好富民强市新征程的重要一年，任务繁重、挑战艰巨。我市地区生产总值仅居成都平原经济区8市第六位，人均GDP仅为全省的77.2%，必须做大经济总量。新兴产业占比小、链条短，传统产业层次低，必须提升发展质量。科技对经济增长的贡献率低于全省平均水平，创新驱动、内生增长的动力仍显不足，必须催生发展新动能。同时，我们也清醒看到加快发展、弯道超车的支撑条件和突破口。近10年探索形成的绿色发展模式是最明显的优势，有利于我市实施绿色引领战略，加快转型升级步伐。全省全面创新改革是最好的契机，有利于我市实施创新驱动战略，加快新旧动力转换接续。地处成渝中心节点的交通区位是最现实的基础，有利于我市实施开放带动战略，加快拓展发展新空间。加速推进的新型城镇化是最大的潜力，有利于我市实施区域联动战略，加快破解城乡二元结构。正在实施的脱贫攻坚战，既是最大的政治责任，更是最大的民生工程和发展机遇，有利于我市补齐全面小康短板。我们将保持战略定力，在新一轮发展中抢占先机、争抓主动，把困难和挑战化为前进动力，把机遇和潜力变为现实优势，在“加快发展、弯道超车”的征程中奋力前行。

今年政府工作总体要求是：全面贯彻党的十八大和十八届三中、四中、五中、六中全会精神，深入落实以习近平同志为核心的党中央治国理政新理念新思想新战略，按照中央、省委、市委经济工作会议和市第七次党代会各项部署，坚持稳中求进工作总基调，以提高发展质量和效益为中心，以推进供给侧结构性改革为主线，以绿色发展为引领，深入实施富民强市“七大提升行动”，统筹抓好稳增长、促改革、调结构、惠民生、防风险各项工作，促进经济平稳健康发展、社会和谐稳定。

今年全市经济社会发展主要预期目标为：地区生产总值同比增长8.5%，全社会固定资产投资增长10%，社会消费品零售总额增长11%，地方一般公共预算收入增长9%，城乡居民人均可支配收入分别增长8.1%、9.5%，节能减排和环境保护完成省政府下达任务。为实现上述目标，我们将突出抓好以下几方面工作。

（一）聚力实体振兴，筑牢经济发展根基。坚持把实体经济作为稳中有进的基石，多措并举激发活力。

创新驱动引领实体经济转型。以全面创新改革试验为牵引，启动国家创新型试点城市、国家知识产权示范城市、国家大众创业万众创新示范基地创建工作。实施高端人才引进、企业家振兴、工匠大师锻造等“遂州英才”七大计划，落实资金补助、股权期权激励等人才强市措施，打造吸引人才的洼地。建设创新示范、知识产权服务等七大平台，支持天齐锂业、英创力、中腾能源等企业创建省级重点实验室或工程技术中心，推进赛伯乐（遂宁）双创中心建设，新增国家高新技术企业10户、军民融合企业3户以上。推进“一提一创一培”，推动8户重点企业开展质量对标提升，新创建12件著名和知名商标，培育新能源轻卡、石墨烯应用产品等新增长点。

降低成本助力企业轻装上阵。引导要素供应企业与实体企业共生共赢，增强要素供给，降低要素成本。稳步推进输配电价改革，落实好富余电、留存电、直购电、“下浮式”阶梯气价等政策，降低能源成本。规范金融机构中间收费，创新金融产品，降低融资成本。推动铁路运输“路企”对接，整顿货运车辆收费，降低物流成本。推行工业用地弹性出让、租让结合，降低用地成本。严格落实减税降费政策，降低制度性交易成本。因企施策、分类指导，鼓励有前景的停产企业实施债务重组、兼并重组，引导产品无市场的停产企业实施破产清算，释放有限资源。

财金互动引导资金脱虚向实。充分发挥财政资金引导作用，安排工业、科技、现代服务业等专项发展资金2.4亿元，用好应急转贷资金和2.55亿元新兴产业创投基金，探索设立首期10亿元的产业发展基金。严格落实财政资金统筹存储制度，按规定逗硬兑现支农小贷奖、企业贷款风险补贴等激励政策，积极支持金融机构扩大信贷规模。鼓励企业通过股权交易、发行企业债、信托、融资租赁等方式拓宽融资渠道，推动英创力、蓝彩电子、飞亚曲轴等企业加快上市步伐。

（二）聚力两化互动，促进产城融合发展。坚持把产业转型升级与新型城镇化有机结合，打造宜居宜业遂宁。

推动制造业向中高端迈进。壮大龙头企业，落实市级领导“一对一”联系重点企业工作机制，完善帮扶措施，力争天齐锂业产值突破50亿元，沱牌舍得产值达到30亿元，磨溪净化厂年处理天然气100亿方，四川江淮汽车下线整车3万辆以上，盛马化工100万吨汽柴油加氢精制装置投运，创维西部产业园电子和纺织基地建成，喜之郎一期全面投产。做强中小企业，搭建企业供需平台，促进龙头企业提高本地配套率，培育省级“小巨人”“专精特新”中小企业30户以上，新增规上工业企业40户以上。筑好产业终端，围绕产业发展规划，强链延链补链，倾力引进OLED电视、动力电池、智能穿戴等终端产品项目。发展智能制造，深入实施“中国制造2025”遂宁行动计划，推动10个智能升级项目和20个智能车间建设。夯实园区平台，提升园区检验检测、产品推广等综合服务能力，力争创建2个省级开发区和1个省级新型工业化产业示范基地。

促进服务业向品质化提升。深入实施服务业“532”工程，力争服务业增加值增长10.5%。围绕增加物流量发展现代物流产业，加快安居长久物流二期、蓬溪粮食物流园等项目建设，建立区域物流公共信息服务平台，探索发展第四方物流，引进培育物流供应链集成商。围绕增加税收留存发展电子商务产业，推进“全企入网、全民触网、电商示范”三大工程，加快电子商务进农村示范县项目建设，支持远能达电商产业园等平台提升服务能力，力争电商交易额增长25%以上。围绕吸引和服务游客发展文化旅游产业，加快五大旅游度假区建设，打造四大精品线路，推动圣莲岛荷博园创建国家4A级景区，培育2个文化创意产业孵化基地，促进旅游与文化、农业、林业、康养等产业深度融合，力争旅游收入增长24.2%。依托区位优势发展会展产业，引进大型会展企业、协会，提升市体育中心、远成物流主题园等场馆会展功能，加快铁路物流园国际会展中心建设。

深化新型城镇化综合试点。突出“三城”建设，疏解老城、发展新区，力争城镇化率提高1.3个百分点。完善城市功能，力争市政设施投资达到40亿元。完成海绵城市试点任务，建成城市地下综合管廊6.14公里。加快国家智慧城市试点建设，完成智慧遂宁云平台一期工程。实施通善大桥升级改造、城区道路平交节点改造等9个“缓堵保畅”工程，推进停车场、充电桩等设施建设，加快城南、北固等公交场站建设，优化红绿灯、标识标线等交通设施设置。统筹城乡发展，推进市、县“多规合一”。加快改造老旧小区，改造棚户区13073户，启动5个花园小镇、15个生态小镇建设，新建成180个幸福美丽新村。深入开展城市品牌创建，争取成功创建全国文明城市、水生态文明城市，加大城市品牌营销力度，提升城市知名度。加快滨江南路、五彩缤纷北路等景观项目建设，打造环观音湖“翡翠项链”，启动观音湖申创国家湿地公园工作。加强遂宁火车站周边、渠河沿线等区域风貌整治。

（三）聚力扩大需求，稳定投资激活消费。在深化供给侧结构性改革的同时，适度扩大需求，合力稳定经济增长。

扭住投资关键作用。继续开展“项目年”活动，完成固定资产投资1238亿元。精准对接国家、省预算内资金和专项建设基金等政策投向，加强项目编制和申报。做实储备项目效益分析、要素保障等前期工作，提高项目计划可靠性。加快120个市级重大项目建设，力争四川蓬船灌区白鹤林水库大坝完成灌浆，唐家渡电航完成河道疏浚，中环线除凤台大桥及其连接线外全线通车，柳树电航竣工。支持社会资本投入医院、养老、污水治理、园区建设等领域，力争完成民间投资680亿元。

增强消费拉动作用。深化“放心舒心消费城市”创建行动，严厉打击制售假冒伪劣产品和网络诈骗、价格欺诈等行为，让群众放心消费。积极参加省级市场拓展“三大活动”，组织开展观音文化节、全国荷花展、全国物流技术装备创新大会、西部金穗粮展会等重大活动。顺应消费升级趋势，提升批零住餐等传统消费，培育教育、信息、文化、康养等消费新热点，新增限额以上商贸企业35户。创新消费方式，鼓励金融机构开发消费信贷产品，支持企业线上线下融合。科学确定城市开发规模、建设时序，引进有实力的开发企业，促进房地产市场平稳健康发展。

发挥引资促进作用。实施“精准招商年”行动，围绕完善产业链、提升价值链，分层分类、定向定点招商。积极参加“2017中外知名企业四川行”等省级重大招商活动，注重发挥异地遂宁商会和在外遂商以商招商作用，力争签约天士力大健康、中车产业园、藏格钾肥动力电池等重大项目，引进投资10亿元以上项目12个。加大签约项目跟踪服务力度，促进项目落地见效，到位市外国内资金450亿元。坚持互惠共赢，规范招商引资政策，统筹招商项目布局，减少同质化竞争，避免无序化竞争。坚持本土企业扶持与招商引资并重，引导本土企业做大做强。

（四）聚力供给优化，转变农业发展方式。坚持农业基础地位不动摇，推进农业供给侧结构性改革，把遂宁农产品好的做多、多的做好，提高农业综合效益。

扩面延链优化产业体系。稳定粮食生产能力，确保粮食总产量在160万吨以上。打造粮油、中药材、畜禽等优势产业带，新建现代农业产业基地20万亩、标准化蔬菜基地3万亩，新改扩建畜禽标准化养殖小区60个。促进农业“接二连三”，将支农资金、土地利用指标向农产品加工等领域倾斜，加快中绿西南基地等5个农业加工项目建设。以“1 5 19”现代农业园区为载体，加快“十里荷画”、天福现代农业旅游度假区等10个乡村旅游项目建设。

提质增效优化生产体系。按照标准化、品牌化、高端化要求，大力发展“三品一标”产品，争创全省农产品质量安全监管示范市。实施“区域品牌 企业品牌”战略，推进农超对接、农批对接，建设“遂宁鲜”营销网点10个以上。提升农业综合生产能力，建成173公里现代农业园区大环线，新建10万亩高标准农田，整治8座病险水库，加快建设“6 2”骨干水利工程。

培育主体优化经营体系。积极培育新型经营主体，新增市级农业龙头企业10家、农民合作社215个，完善与农户利益联结机制。依法依规、适度规模流转土地，建立土地流转保证金制度，预防和妥善解决改变土地用途、拖欠租金及土地撂荒等问题。加强农事服务超市建设，完善农业服务体系。深化与高等院校、科研单位合作，设立川农大新农村研究院遂宁分院。

（五）聚力改革开放，持续增强内生动能。坚持以改革破除发展障碍，以开放拓展发展空间。

以枢纽提升聚优势。巩固铁路干线枢纽地位，争取成遂南达高速铁路列入国家“十三五”铁路网规划，力争绵遂内城际铁路、遂宁动车运用所达到开工条件，完善遂宁火车站服务设施，促进公铁联运。加快区域路网建设，推动成南高速扩容和遂德等高速公路建设，实施3条国道、4条省道改造。提高市域联通效率，建成遂西高速吉祥连接线、遂宁至大英快捷通道，启动涪江六桥规划，新改建乡镇联网公路100公里、村联网路120公里。加快立体交通枢纽建设，力争机场迁建项目开工，涪江复航前期工作取得突破性进展。

以区域合作拓空间。加快成遂协同发展进程，探索遂宁开发区与成都高新区共建产业园区，强化与重庆协作配套发展。加强与德阳、绵阳在装备制造、新能源、新材料、信息技术等领域的合作，启动建设欧美产业园。加快建设物流大通道，与西安、宁波、临沂等地建立合作机制，支持珠海港驻遂宁办事处做大海铁联运业务。推进国际陆港城市申建工作，推动电子口岸信息化建设，积极融入“全国通关一体化”。组织办好“第八届泛成渝经济区商会合作峰会暨首届遂商大会”。支持遂宁银行跨区域发展，实施“千亿企业创建工程”。

以改革攻坚激活力。扎实推进各项改革试点，加快实施已出台的71项改革方案。深化城管体制、环保执法管理等行政管理体制改革。支持射洪撤县建市。启动全省“多证合一”试点示范城市创建工作。完善市区分税制财政体制，探索建立市以下转移支付制度，加强政府债务管理。细化落实“三权分置”办法，深化农村产权制度改革。稳妥发展混合所有制经济，完善市属国企法人治理结构。深化出租汽车行业改革，规范发展网约车。

（六）聚力脱贫攻坚，补齐全面小康短板。围绕“两不愁、三保障”和“四个好”目标，推进精准扶贫，强化稳定脱贫，确保2.5万贫困人口脱贫、90个贫困村摘帽。

加强产业就业扶持。用好贫困村产业扶持基金，推广“四六开”“股权分红”“土地入股”等模式，贫困村优质粮油、果蔬面积达到18.5万亩以上，出栏家畜23.3万头。加强职业技能培训，在镇村开发公益性岗位，力争有劳动能力的贫困家庭至少1人就业。壮大教育扶贫救助基金，落实贫困学生15年基础教育免学费等相关政策，推动贫困区域70所学校标准化建设，选派100名优秀教师轮岗支教。

深入推进健康扶贫。全面落实医疗扶助政策，努力解决因病致贫、返贫问题。开展中医药能力提升、医疗专家支医、村医能力提升、分类分批救治“四大工程”，提高医疗服务水平。强化家庭医生、健康管理等公共卫生服务，提高贫困人口健康水平。今年拟退出贫困村实现标准化卫生室、合格乡村医生“两个全覆盖”。

改善生产生活条件。新建集中供水工程52处，新增和恢复灌溉面积4.15万亩。完成4000户贫困户危房改造、10471名贫困人口易地扶贫搬迁。贫困地区广播电视数字化覆盖率达到100%。通过低保线与扶贫线“两线合一”、特困人员救助等途径，保障无劳动能力、无生活来源、无法通过产业扶持和就业帮助的贫困人口基本生活。

完善工作推进机制。强化精准扶贫管理，优化项目安排、资金使用、驻村帮扶等工作流程，进一步扶准扶好。加大扶贫资金投入，财政专项扶贫预算资金增幅高于一般公共预算收入增幅30%以上。整合涉农资金，引导更多金融资金、民间资本投入扶贫领域。强化社会扶贫“五大平台”功能，营造良好氛围。落实“三个一”调度机制，及时解决问题和困难。

（七）聚力民生改善，不断增进民众福祉。坚持把人民群众对美好生活的向往作为奋斗目标，做到民生投入只增不减、为群众办实事只增不减，让人民群众更有获得感。

围绕“保障实”加大民生投入。继续实施民生工程和民生大事，一般公共预算民生支出占比稳定在69%左右。深入推进全民参保登记，全面实行五险统一征收和稽核，妥善解决被征地农转非人员参加养老保险问题，探索医保市级统筹。城乡最低生活保障补差标准增幅不低于省上规定。城镇新增就业3.3万人。关爱老年人和困境儿童，依法保障妇女、未成年人、残疾人权益，加强特殊人群服务管理。

围绕“生活好”均衡公共服务。实施义务教育学校标准化建设，改造校舍7.1万平方米，大英通过国家义务教育均衡发展验收，安居、蓬溪通过省义务教育均衡发展验收。优化义务教育学校布局，新建、改扩建河东二小等12所公办学校，强化居民小区配套幼儿园建管，大力发展公办幼儿园。实施乡村教师支持计划，推进名师名校长工程，提升公办高中质量，加快现代职教体系建设，鼓励社会力量提供多样化教育服务。完善分级诊疗配套政策，促进基层首诊。开展12个大项基本公共卫生服务，探索全民大健康体检机制，推进省级健康城市试点建设。健全医疗费用控制定期通报、处方点评等制度，整治过度医疗等行为。鼓励民营和公立医院办养老机构，提高社区老年人活动中心建设标准。健全公共文化、全民健身服务体系，启动“高清遂宁智慧广电”建设，加快宋瓷文化中心、卓筒井大遗址公园规划建设，举办遂宁市第六届运动会。

围绕“环境美”厚植生态优势。实施生态惠民工程，建设国家生态文明先行示范区。深化“七大绿化工程”，推进天然林资源保护和退耕还林。打好大气、水、土壤污染防治“三大战役”，做好环保督察工作，严惩环境违法行为。实施“减排、压煤、抑尘、治车、控秸”五大工程，让群众少些“霾怨”。全面落实河长制，对全市6条重要河流、城区5条河流，市县乡村分级分段设立河长，全力优化河湖生态。整治城镇黑臭水体，实施渠河饮用水源取水点集中北移工程。实施“化肥农药零增长行动”，加强农业面源污染、重金属污染防治。

围绕“秩序优”创新社会治理。全面从严落实安全生产责任，持续开展重点领域专项整治，深化隐患排查治理，坚决遏制重特大事故发生。健全五级食品药品监管体系，推进“明厨亮灶”，规范网络订餐管理等工作。深入开展“法律七进”活动，完善网格化管理机制，大力创建和谐社区。积极化解民间借贷、征地拆迁等领域矛盾纠纷。构建立体化社会治安防控体系，完成220个幸福美丽新村“雪亮工程”，严厉打击违法犯罪活动。支持工会、共青团、妇联等群团组织和社会组织参与社会治理，构建多元共治格局。强化应急管理，做好人感染H7N9流感防控等应急处置工作。加强国防动员、优抚安置、双拥、对台、侨务和外事等工作。

三、始终崇尚实干，干在当下成在实处

各位代表，“加快发展、弯道超车”新使命对政府自身建设提出了新的更高要求。我们要始终担当在肩，务实重行、奋发有为，不断增强政府执行力和公信力，建设人民满意政府。

（一）坚持简政放权，转变职能抓落实

让“放”更有含金量。强化权力清单和责任清单动态调整及后续管理，推动“两张清单”向基层延伸。推进船山、射洪相对集中行政许可权改革试点，逐步实现“一枚公章管审批”。

让“管”更有震慑力。全面推行“双随机一公开”，促进监管工作标准化、规范化。加强诚信体系建设，落实举报奖励、“黑名单”等制度，推动建立跨部门联合“失信惩戒机制”。在相对集中行政许可权改革试点中坚持审管分离，加强事中事后监管。

让“服”更有获得感。持续提升发展软环境，营造良好“亲”“清”政商关系。优化审批服务流程，全面推行“一窗办理”“互联网＋政务服务”，促进便民服务事项向基层下沉。清理取消涉及群众办事的不合理证明和手续，让群众办事更便捷。

（二）坚持依法行政，规范用权抓落实

尊重程序履好职。深入实施法治政府建设实施方案，完善政府法律顾问制度，把好行政决策合法性审查、集体讨论等关口。做好《遂宁市城市管理条例》立法调研、起草相关工作。完善行政决策终身责任追究及责任倒查机制。

公正文明执好法。在交通运输、规划建设、农业等领域探索综合执法改革，避免多头执法。结合扩权强镇试点，促进执法力量向乡镇下沉。健全行政执法与刑事司法衔接机制，维护执法严肃性、权威性。

接受监督用好权。自觉接受人大法律监督、工作监督和政协民主监督，认真办理人大议案、人大代表建议和政协委员提案。主动接受舆论监督和社会公众监督，深入开展“阳光问廉”活动，继续办好“12345”政府服务热线。加强和改进行政复议，提高行政机关负责人出庭应诉率。

（三）坚持务实勤政，提升效能抓落实

工作推进“项目化”。围绕“七大提升行动”工作方案，梳理重大工作推进流程、关键节点，实行定量、定性、定岗、定责管理。构建环环紧扣、责任清晰的执行链条，做到挂图作战、对表作业。

聚焦执行“大督查”。整合督查、监察、效能等力量，构建大督查格局。围绕重大决策、重大项目等重点领域，建立督查台账，盯住重点岗位、重点环节全程督查。坚持明查暗访相结合，掌握工作真实情况。

绩效管理“差异化”。科学合理分类考评对象，健全差异化考核指标体系。完善第三方绩效评价办法，让考评结果更加客观公正。拉大考核等次差距，激发干部队伍活力。

（四）坚持挺纪在前，改善政风抓落实

坚守纪律生命线。牢固树立“四个意识”，自觉遵守政治纪律和政治规矩。常态化推进党纪党规学习教育，拧紧思想“总开关”。坚持失责必问、问责必严，以常态化问责增强责任意识和担当意识。

树好作风风向标。持续纠正“四风”，精简各类文件，改进会议形式。践行群众路线，完善现场调研、民情访谈等制度，及时解决群众诉求。落实正向激励和容错纠错机制，大力整治不作为、乱作为、慢作为现象。

夯实廉政防火墙。压紧压实主体责任，严格落实“一岗双责”，完善廉政风险防控机制，加强招投标、公共资源交易等重点领域监管。强化行政监察和审计监督，严查重处违纪违法行为。

各位代表，“众志开新篇，实干铸辉煌”。让我们在省委、省政府和市委的坚强领导下，务实重行，拼搏进取，扎实推进富民强市“七大提升行动”，以优异成绩迎接党的十九大和省第十一次党代会胜利召开！
